# Effect of three rehabilitation methods combined with transcranial electromagnetic stimulation on post-stroke aphasia: a RCT network meta-analysis

**DOI:** 10.3389/fneur.2025.1600065

**Published:** 2025-05-27

**Authors:** Xinyu Lin, Haojie Li, Xie Wu, Rui Huang

**Affiliations:** School of Exercise and Health, Shanghai University of Sport, Shanghai, China

**Keywords:** aphasia, stroke, neurological rehabilitation, combined rehabilitation approach, cervical cranial electrical stimulation

## Abstract

**Background:**

Stroke is a leading cause of death and disability worldwide, particularly in China, where it affects younger populations. Aphasia, a common post-stroke disorder, impairs language skills and occurs in 30–40% of stroke patients. Neuromodulation techniques like transcranial magnetic stimulation (TMS) and transcranial direct current stimulation (tDCS) have shown promise in aphasia rehabilitation. Combining these methods with traditional treatments may improve recovery and shorten rehabilitation time. This study examines the effectiveness of these combined therapies in post-stroke aphasia (PSA) to inform clinical practice.

**Methods:**

Six databases, including PubMed, Embase, and Web of Science, were systematically searched, and 14 randomized controlled trials (RCTs) with 376 stroke patients were finally included. The outcome indicators were aphasia and quality of life related indicators. Net meta-analysis was performed using Stata 17.0 to assess the relative effectiveness of each combined intervention and to test the consistency of direct and indirect evidence.

**Results:**

In this study, a total of 14 randomized controlled trials (RCTs) involving 376 patients with stroke were included. For the primary outcome metrics, SLT was most effective in improving Naming (SMD = 1.09, 95% CI [0.16, 2.02], *p* < 0.05, [SUCRA] = 85.2). comprehensive speech and language therapy (CSLT) was most effective in improving Comprehension in stroke patients (SMD = 1.01, 95% CI [0.22, 1.80], *p* < 0.05, [SUCRA] = 84.5). CSLT (SMD = 0.83, 95% CI [0.07, 1.58], *p* < 0.05, [SUCRA] = 74.0) and SLT (SMD = 0.87, 95% CI [0.13, 1.61], *p* < 0.05, [SUCRA] = 76.4) better in improving Repetition in stroke patients.

**Conclusion:**

SLT and CSLT can effectively improve aphasia in stroke patients. It is recommended to prioritize their application in clinical rehabilitation.

**Systematic review registration:**

https://www.crd.york.ac.uk/prospero/, identifier CRD42024611829.

## 1 Introduction

Stroke is a leading global cause of death and disability, burdening patients and families (1, 2). With global population aging, its incidence is rising, even among younger people, and in China, unhealthy lifestyles like hypertension, diabetes, smoking, and drinking contribute to a younger—onset trend (3). Stroke patients often have multiple impairments such as motor, sensory, swallowing, and speech dysfunctions (4, 5). Motor and speech impairments most affect independent living and social function; speech dysfunction in particular impacts communication, mental state, social interaction, and work ability (6). Thus, post-stroke rehabilitation is crucial for function recovery and reducing disability and death risks.

Post-stroke Aphasia (PSA) is one of the more common neurocognitive disorders in stroke patients, which is mainly characterized by impaired speech comprehension, expression, reading and writing (7). The occurrence of aphasia is usually closely related to the damage of the language function area of the brain (8). There are various types of aphasia, and the common ones include expressive aphasia, comprehension aphasia, naming aphasia, and mixed aphasia (9). According to clinical research, about 30%−40% of stroke patients will have different degrees of aphasia, and among them, severe aphasia will cause patients to completely lose their language communication ability, which will affect their daily life and social function (10). Studies have shown that (11) aphasia not only affects the patient's language ability, but may also lead to the exacerbation of emotional problems, such as depression and anxiety. Kao and Chan's (12) study showed that emotional disturbances in patients with aphasia were significantly correlated with speech dysfunction, and the presence of aphasia significantly increased the psychological burden of stroke patients. In addition, patients' inability to communicate effectively with others due to language impairment may lead to social isolation and estrangement of family relationships. Early rehabilitation and treatment targeting aphasia is essential to minimize these negative effects. Further studies have also shown that restoration of language function has a positive effect on the quality of daily life, social interaction, and self-care ability of patients (13).

Transcranial magnetic stimulation (TMS) and transcranial direct current stimulation (tDCS), non-invasive neuromodulation techniques, are widely applied in neuroscience. TMS uses brief magnetic field pulses to act on the cerebral cortex, altering brain electrical activity, promoting neuroplasticity, and having the potential to improve neurological function (14). tDCS, on the other hand, employs weak direct currents to regulate neuronal excitability and synchronization for brain function restoration (15). Numerous recent studies have proven the efficacy of transcranial electromagnetic stimulation in treating post-stroke sequelae, particularly in aphasia rehabilitation. Sheng et al. (16) showed that rTMS can effectively boost language function recovery in stroke patients, especially when Broca's area is damaged, enhancing speech production. tDCS has also demonstrated therapeutic potential for language disorders, improving speech fluency and comprehension (17). Nevertheless, these techniques have limitations. TMS demands high patient compliance, and while tDCS is easy to administer, its efficacy can be influenced by individual differences, and the treatment duration is often long.

Currently, post-stroke aphasia rehabilitation mainly involves speech therapy, cognitive training, and neuromodulation techniques. Speech therapy, the most common treatment, improves patients' verbal expression and language comprehension via language, hearing, and communication skills training (18). It effectively aids in partial language function recovery, especially for mild–to–moderate aphasia patients. Yet, it requires long-term adherence, has an intensive process, and its efficacy varies due to individual differences (19). Recently, emerging neurorehabilitation methods like virtual reality (VR) therapy and computer—assisted training have been applied to aphasia rehabilitation. These offer richer interactive experiences to promote neuroplasticity and recovery, but they mainly focus on enhancing speech ability without directly modulating brain or neurological functions. Thus, integrating these treatments with more effective neuromodulation techniques has become a key research area in rehabilitation.

While many studies focus on single rehabilitation or neuromodulation for post-stroke aphasia, combined treatment research is scarce. Combined therapy, which combines advantages of different methods, may improve treatment, shorten cycles, and boost patient compliance. Specifically, combining transcranial electromagnetic stimulation with traditional rehab can enhance language recovery via brain electrical activity regulation and neuroplasticity promotion. This study innovatively combines three common rehab methods with repetitive transcranial electromagnetic stimulation to explore its efficacy for post-stroke aphasia. Network meta-analysis of RCTs synthesizes results to assess the combined treatment, offering a new clinical basis. Given the limited number of experiments and efficacy differences in prior studies, this research aims to provide stronger evidence for future practice and new ideas for personalized aphasia treatment.

## 2 Methods

This study was guided by the Preferred Reporting Items for Systematic Evaluation and Meta-Analysis (PRISMA list of NMAs10 and the Cochrane Handbook for the Evaluation of Intervention Systems). Registration number: CRD42024611829.

### 2.1 Data sources

We conducted systematic searches in PubMed, Embase, Web of Science, Cochrane, EBSCO, and China National Knowledge Infrastructure (CNKI), and the selection of included studies was done independently by 2 researchers (XL, HL). Searches were performed in PubMed and Cochrane using terms in MeSH. Searches were performed in Embase using terms in Entree and in CNKI using subject terms combined with free terms. The reference lists of relevant articles were also manually screened for other studies that might be eligible. The time frame of the search was from January 2000 up to September 2024, and it was limited to human studies published in Chinese or English, and only core journals were included in Chinese.

The search strategy followed the PICOS principles of evidence-based medicine: (P) population: stroke patients with aphasia; (I) intervention: comprehensive speech and language therapy (CSLT), language cognitive therapy and speech and language therapy combined with transcranial electromagnetic stimulation; (C) Control group: non-combination therapy; (O) Outcomes: including ratings of naming, spontaneous speech, listening comprehension, repetition, AQ and quality of life; (S) Study type: RCTs.

### 2.2 Study selection

**Figure 1:**
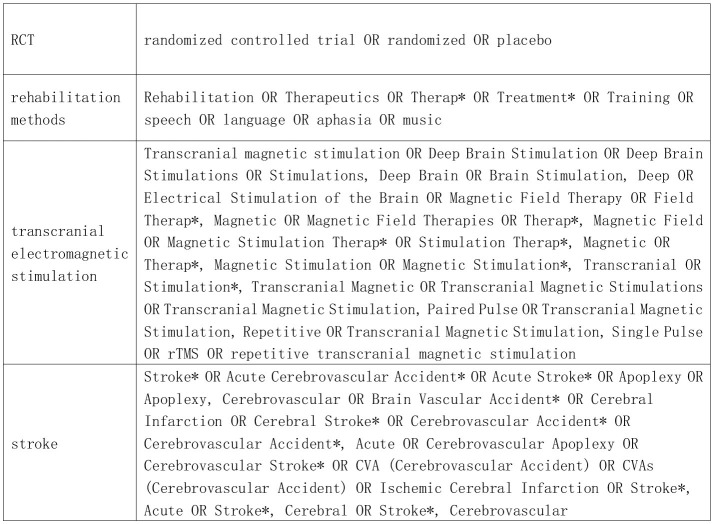
Search strategy.

The obtained literature was screened. Duplicate items were first eliminated by endnote automatic weight checking, and then duplicate literature was manually removed by reading the headings. The remaining literature was further screened to eliminate non-stroke disease studies, studies that did not assess cognitive function or negative mood, studies without repetitive transcranial magnetic stimulation, studies that were not in the combined category, reviews, conference abstracts, animal studies, study protocols, case reports, retrospective studies, and book chapters. The search strategy is shown in Figure 1.

### 2.3 Eligibility criteria

We included randomized clinical trials in people with confirmed acute or chronic stroke (included randomized clinical trials in people with) comparing the effects of different rehabilitation methods combined with transcranial electromagnetic stimulation vs. a no combined group.

Studies that met the following criteria were eligible for inclusion: (1) they were RCTs; (2) acute or chronic stroke patients with aphasia; (3) it was a certain rehabilitation method combined with transcranial electromagnetic stimulation; (4) data on outcome indicators were complete; and (5) the intervention in the experimental group was COMPREHENSIVE SPEECH AND LANGUAGE THERAPY, language cognitive therapy and speech and language therapy combined with transcranial electromagnetic stimulation in the experimental group, and the control group underwent some kind of rehabilitation method combined with sham rTMS intervention; (6) measuring at least one of the indicators: naming, spontaneous speech, listening comprehension, repetition, AQ and quality of life.

Studies were excluded if they: (1) were non-RCTs; (2) were animal experimental studies, review-type literature, conference reports, case reports, letters, and repetitively published literature, etc.; (3) full text was not available; (4) experimental results data were incomplete or data indicators could not be extracted; (5) relevant indicators of interest to this study were not reported; (6) patients had other neurological diseases besides stroke; (7) Non-core journal literature in Chinese published literature.

### 2.4 Data collection

The collected literature was imported into EndNote 20 software by 2 researchers (XL, HL) according to the search strategy and the obtained literature was screened. Duplicate literature was first excluded, and then titles and abstracts were read for initial screening. The remaining literature was further screened by reading the full text in detail according to the inclusion and exclusion criteria. Subsequently, 2 researchers (XL, HL) cross-checked the results of their respective screening, and if the checking was consistent, the study was included; if there was any disagreement, the third researcher (WS) was consulted and final inclusion was made after discussion and agreement.

For eligible trials, 2 trained researchers (XL, HL) independently extracted data from the included literature using a standardized data extraction form and generalized the risk of risk bias. The extracted data mainly included (1) basic information about the included literature (first author, year of publication, country, etc.); (2) demographic characteristics of the subjects (number of experimental and control groups, age, sex, and duration of illness); (3) details of the interventions (type of intervention, intensity, duration, and frequency); and (4) outcome metrics (mean and standard deviation, with the primary outcome metrics selected including scales for rating naming, understanding, and repetition); the primary outcome metrics selected included scales for assessing naming, understanding, and repetition; and the primary outcome metrics selected included scales for assessing the risk of risk bias. The primary outcome indicators selected included scales rating naming, comprehension, and repetition; the secondary outcome indicators selected included scales rating spontaneous speech, AQ, and quality of life. For studies in which results were presented graphically without numerical summaries, numerical data were extracted for analysis using a validated plot digitizing tool (GetData 2.22). We contacted the authors of the articles for information when necessary.

### 2.5 Risk of bias of the systematic review

All eligible studies were assessed for risk of bias (ROB) by 2 researchers (XL, HL) according to the Cochrane 5.1 version of the Risk of Bias Assessment Tool (which includes seven domains (random sequence generation, allocation concealment, blinding of participants and personnel, blinding of outcome assessor, incomplete data outcome, selective reporting, and other bias), 2 researchers (XL, HL) assessed risk of bias (ROB) for all eligible studies. Risk assessment analyses were performed using Review Manager 5.3 (Scandinavian Cochrane, Denmark), and each area was assessed as unclear, low risk, and high risk. Based on these assessments, we categorized the overall risk of bias for each study as (1) low ROB: there were no domains assessed as high risk, and there may have been domains assessed as unclear but fewer than three; (2) medium ROB: there was a domain that was assessed as high risk but no more than one; or there were no high risk domains but more than three domains that were assessed as unclear; and (3) high ROB: all cases other than the above are categorized as high risk.

### 2.6 Statistical analysis

In this study, data were analyzed by META using STATA 17.0 software (Stata Corp LLC, College Station, TX, USA), and outcome indicators were continuous variables. This NMA integrated the before and after changes in the experimental and control groups to systematically assess the effects of different rehabilitation methods combined with transcranial electromagnetic stimulation on aphasia after stroke, and to accurately assess the effects of these interventions, we calculated standardized mean differences (SMDs) and their 95% confidence intervals (CIs) for each indicator, with a uniformly adjusted baseline of α = 0.05, and combined effect estimates based on a random effects model to address heterogeneity between studies in terms of participant characteristics and intervention modalities. Heterogeneity was quantified using the *I*^2^ statistic and Cochran's Q-test. The relationship between different rehabilitation methods combined with transcranial electromagnetic stimulation was visualized by means of a network diagram, where the lines connecting the nodes represent direct comparisons between different rehabilitation methods combined with transcranial electromagnetic stimulation. The size of the nodes and the thickness of the connecting lines are proportional to the number of studies that included that comparison, and this graphical presentation visualizes the relative strength of each intervention and its position in the network. In addition, the plotted network contributions further quantify the contribution of each direct comparison to the overall network, helping to analyze the influence of each intervention across the network. Additionally, to assess publication bias in the study, corrected comparison funnel plots were used to analyze publication bias for the primary outcome metrics. Finally, the probability of being the best intervention was calculated using a cumulative lower surface of the ranking curve (SUCRA) approach.

### 2.7 Three rehabilitation methods

Comprehensive Speech and Language Therapy (CSLT) is an integrated speech and language therapy that emphasizes multimodal and multidimensional rehabilitation strategies. It not only includes traditional speech therapy but also incorporates a variety of advanced treatment methods, such as Constraint-Induced Language Therapy (CILT), Music Therapy (MT), Melodic Intonation Therapy (MIT), Intensive Language-Action Therapy (ILAT), and Multimodal Aphasia Therapy (M-MAT). These methods, when applied in combination, aim to comprehensively enhance patients' language functions and communication abilities.

Speech and Language Therapy (SLT) is a commonly used conventional speech therapy in clinical practice, primarily focusing on improving patients' speech and language abilities through standardized language training and rehabilitation exercises. This method typically includes phonation training, language comprehension training, and oral expression training, aiming to help patients restore and improve their language functions through systematic training.

Language Cognitive Therapy (LCT) is a rehabilitation method that adds cognitive training to traditional speech therapy, emphasizing the synergistic rehabilitation of language and cognitive functions. It includes Simultaneous Naming Training (SNT), Behavioral Naming Therapy (BNT), Word Association Navigation Training (WANT), and Response Expansion Training (RET). These methods, by integrating cognitive strategies, not only improve language functions but also enhance patients' cognitive abilities and overall communication capabilities.

## 3 Result

### 3.1 Study selection

**Figure 2:**
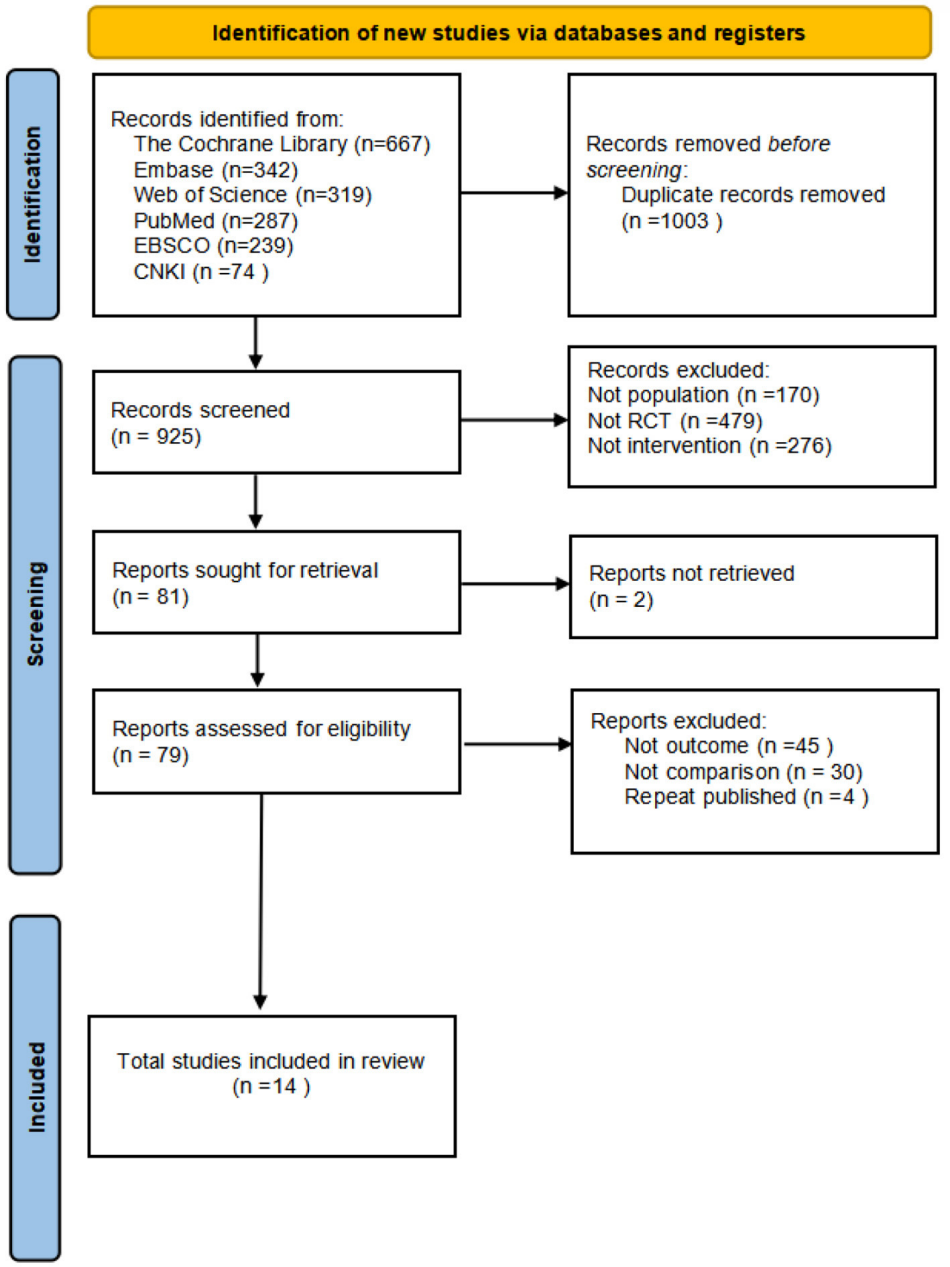
Literature search flowchart.

The flowchart for study selection is shown in Figure 2. A total of 1,928 articles potentially eligible for the study were collected from different databases. To ensure the accuracy of the study and to avoid double counting of the same content, 1,003 duplicate articles were removed through automation and checking. The remaining 925 articles needed to be screened. By analyzing the title and abstract of each article, 844 ineligible articles were deleted to ensure that only the most relevant literature to the study's objectives was retained. Seventy-nine full-text articles were obtained and read, and their study design, sample size, methodological quality, and results were assessed in detail, resulting in the identification of 14 randomized controlled trials (RCTs). These trials were conducted up to September 2022 and all met the quality criteria set by the Institute. Three different rehabilitation treatments were evaluated. Each step of the screening process followed a strictly standardized procedure to ensure the reliability and scientific validity of the results.

### 3.2 Features of the included studies

Fourteen studies were finally included, and the basic characteristics of all included studies are detailed in Table 1. These studies were published between 2013 and 2023 and were conducted in China, Poland, Finland, Spain, and South Korea. A total of 376 stroke patients were included in this study, 187 in the experimental group and 189 in the control group. Demographic data reported included country, age, gender, and disease duration. Rehabilitation methods included CSLT, LCT, and SLT. The mean duration of treatment for the interventions of different rehabilitation methods was 3.1 weeks, and 53.85% of the studies reported that the interventions lasted more than 2 weeks.

**Table 1:** Basic features of the included studies.

**Study**	**Country**	**Group**	**Size (F/M)**	**Age (mean ±SD)**	**Days from onset (mean ±SD)**	**Intervention category**	**Duration**	**Intervention frequency**	**Outcome**
Zhou et al. (25)	China	CON	10 (3/7)	53.3 ± 10.3	71.7 ± 32.7	CSLT	10 days	CSLT:30 min, once/day	WAB, CADL
		CSLT	10(3/7)	51.1 ± 12.2	78.6 ± 33.7	rTMS + CSLT		CSLT:15 min, once/day; rTMS: 20 min, once/day	
Heikkinen et al. (26)	Finland	CON	8 (2/6)	61 ± 14.1	1,440 ± 1,800	CSLT + sham rTMS	2 weeks	CSLT: 3 h, once/day, 5 times/week	WAB
		CSLT	9(2/7)	54 ± 26.7	1,020 ± 1,089	CSLT + rTMS		rTMS: 20 min, once/day, 5 times/week	
Yan et al. (27)	China	CON	13 (3/10)	55.5 ± 12.2	92.2 ± 122.4	sham tDCS + CSLT	2 weeks	CSLT: 30 min, once/day, 5 times/week	WAB, ANT
		CSLT	13(8/5)	63.5 ± 10.2	241.2 ± 373.8	tDCS + CSLT		tDCS: 20 min, once/day, 5 times/week	
Low et al. (28)	Canada	CON	10 (2/8)	63.8 ± 5.6	876 ± 511	CSLT + sham rTMS	2 weeks	CSLT: 3.5 h, once/day, 5 days/week	BNT
		CSLT	10(3/7)	61.5 ± 12.2	1,168 ± 876	CSLT + rTMS		rTMS: 20 min, once/day, 5 times/week	
Liu et al. (29)	China	CON	20 (6/14)	49.5 ± 35.3	54.5 ± 54.8	CSLT	3 weeks	CSLT: 30 min, once/day,5 times/week	WAB
		CSLT	20(5/15)	54.9 ± 16.8	27.0 ± 59.5	rTMS + CSLT		rTMS: 20 min, once/day, 5 times/week	
Wang et al. (30)	China	CON	15 (2/13)	60.4 ± 11.9	483 ± 219	sham rTMS + LCT	2 weeks	LCT: 20 min, once/day,5 times/week	CAAT
		LCT	15(1/14)	61.3 ± 13.2	504 ± 192	rTMS + LCT		rTMS: 20 min, once/day, 5 times/week	
Zhang et al. (31)	China	CON	8	44.9 ± 9.4	228.9 ± 138	sham tDCS + LCT	10 days	LCT: 20 min, twice/day	WAB
		LCT	8	46.9 ± 16.6	168.9 ± 156.3	tDCS + LCT		tDCS: 20 min, twice/day	
Qiu et al. (32)	China	CON	20 (1/19)	55.0 ± 10.7	127.2 ± 51.6	rTMS	4 weeks	LCT: 30 min, once/day, 5 times/week	WAB, CADL
		LCT	20(5/15)	51.1 ± 14.8	50.4 ± 57.9	LCT + rTMS		rTMS: once/day, 5 times/week	
Zheng et al. (33)	China	CON	15 (7/8)	53.3 ± 14.8	50.2 ± 18.5	LCT	4 weeks	LCT: 30 min, once/day, 5 times/week	ABC, MBI
		LCT	15(4/11)	50.5 ± 13.9	52.6 ± 18.1	rTMS + LCT		rTMS: 20 min, once/day, 5 times/week	
Cid-Fernández et al. (34)	Spain	CON	5 (2/3)	59.8 ± 14.4	/	sham tDCS + LCT	2 weeks	LCT: 60 min, once/day, 5 times/week	BDAE, LCI
		LCT	5(2/3)	62.8 ± 16.4	/	tDCS + LCT		tDCS: 20 min, once/day,5 times/week	
Seniów et al. (35)	Poland	CON	20 (10/10)	59.7 ± 10.7	39.9 ± 28.9	SLT + sham rTMS	3 weeks	SLT: 45 min, once/day, 5 times/week	WAB
		SLT	20(8/12)	61.8 ± 11.8	33.5 ± 24.1	SLT + rTMS		rTMS: 30 min, once/day, 5 times/week	
Yoon et al. (36)	Korea	CON	10 (3/7)	61.1 ± 8.7	156 ± 80.1	SLT + sham rTMS	4 weeks	SLT: 60 min, once/day, 5 times/week	K-WAB
		SLT	10(2/8)	60.5 ± 9.6	204 ± 71.7	SLT + rTMS		rTMS: 20 min, once/day, 5 times/week	
Bai et al. (37)	China	CON	10	45.3 ± 6.8	90 ± 45	SLT + sham rTMS	4 weeks	SLT: 20 min, once/day, 5 times/week	WAB
		SLT	10			SLT + rTMS		rTMS: 20 min, once/day, 5 times/week	
Du et al. (38)	China	CON	25 (7/16)	63.6 ± 9.7	35.0 ± 30.7	SLT + sham iTBS	4 weeks	SLT: 30 min, once/day, 6 times/week	WAB, BNT
		SLT	22(13/9)	58.9 ± 12.8	40.0 ± 28.8	SLT + iTBS		iTBS: 200 s, once/day, 6 times/week	

Regarding the reported outcome indicators, those from WAB, ABC, CADL, and CAAT that reflect naming, comprehension, repetition, spontaneous, AQ or quality of life were selected.

### 3.3 Risk of bias assessment

**Figure 3:**
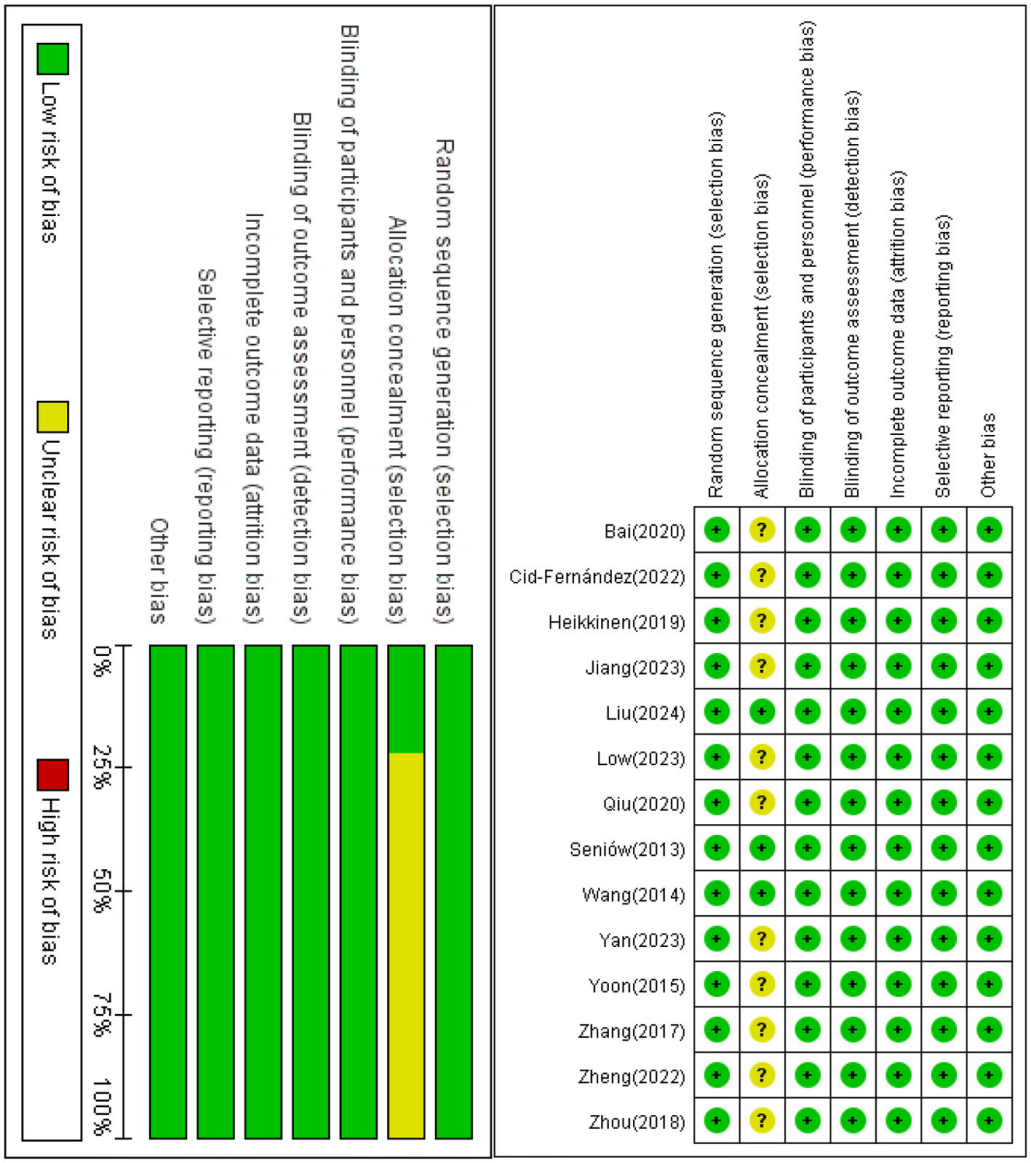
Evaluation results of literature quality risk bias of included studies.

A comprehensive assessment of the risk of bias (ROB) for each study was conducted using the Cochrane Risk of Bias Assessment Tool (RoB 2.0). The following provides in—depth details of this assessment. Among the 14 articles under review, all 14 made references to random allocation. Notably, 8 of them specified the exact method of randomization employed. Regarding allocation concealment, 3 articles clearly stated its implementation. All 14 articles reported on blinding, with a focus on the blinding of outcome assessment, which was also reported in all of these studies. Additionally, all 14 studies demonstrated a low risk of selective reporting, and no other biases were identified across all the articles. In conclusion, after a meticulous evaluation, all 14 articles were determined to have a low ROB. For a more detailed visualization of the literature quality assessment results, please refer to Figure 3.

### 3.4 Direct pairwise meta-analyses

#### 3.4.1 Primary outcome

**Figure 4:**
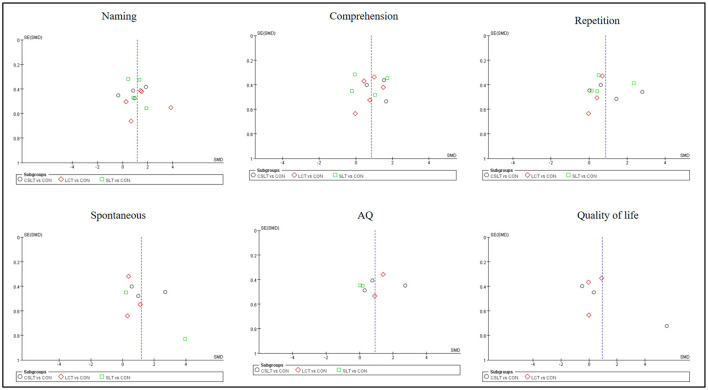
Funnel plot of naming, comprehension, repetition, spontaneous, AQ and quality of life in pairwise meta-analysis.

**Figure 5:**
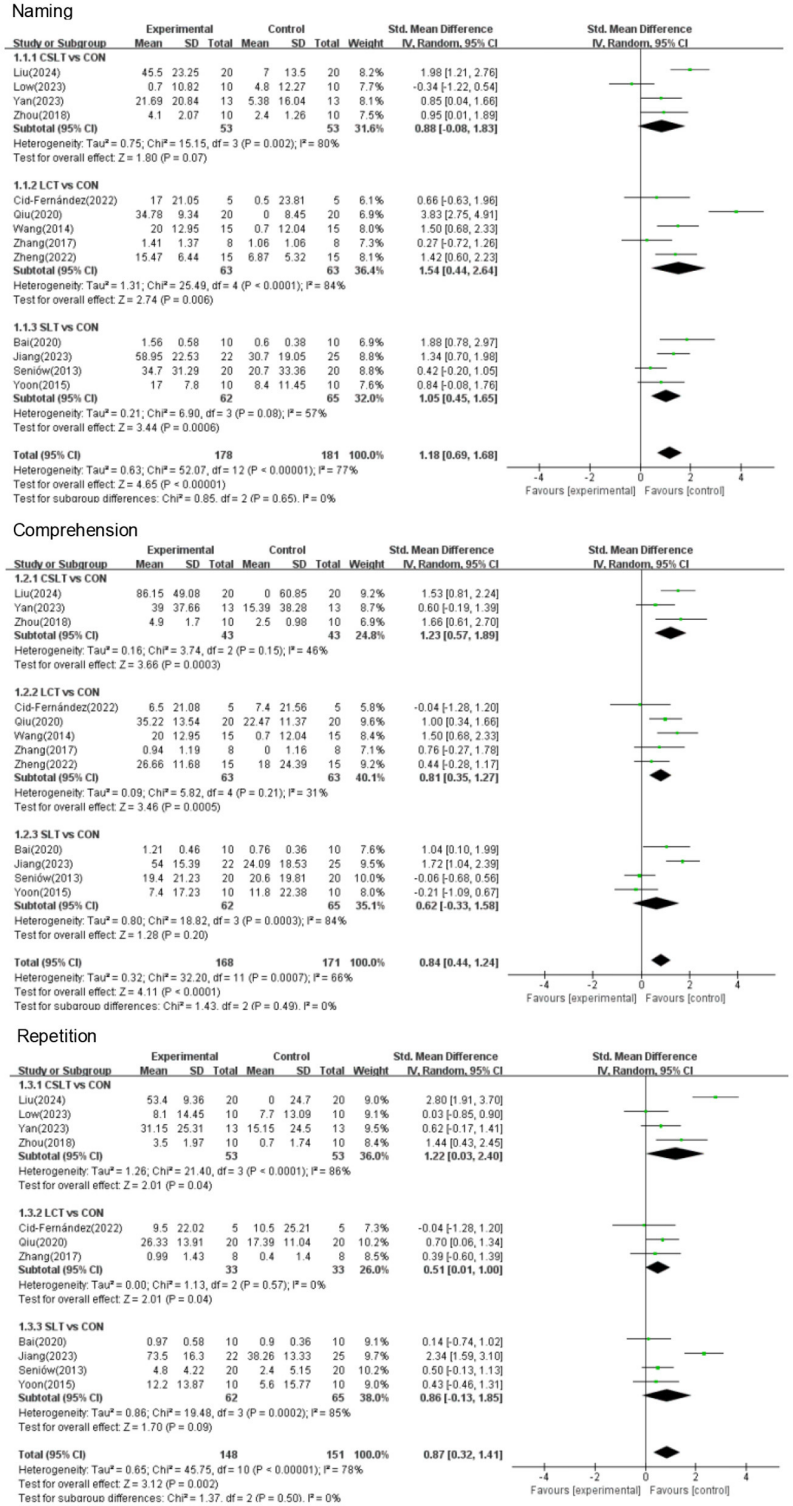
Forest plot of primary outcome.

This NMA began with a two-by-two meta-analysis (Figure 4) and provided forest plots based on the effects of different rehabilitation methods combined with transcranial electromagnetic stimulation on Naming, Comprehension and Repetition (see Figure 5).

CSLT showed a non-significant effect on improving Naming compared to the control group (SMD = 0.88, 95% CI [−0.08, 1.83], *p* > 0.05, *I*^2^ = 78%) with a high degree of heterogeneity, suggesting some variability in the results of the studies. LCT showed a significant effect on improving Naming compared to the control group (SMD = 1.54, 95% CI [0.04, 2.64], *p* < 0.05, *I*^2^ = 84%), suggesting differences in results across studies. SLT similarly showed a significant effect on improving Naming (SMD = 1.05, 95% CI [0.45, 1.65], *p* < 0.001, *I*^2^ = 57%) suggesting a moderate degree of variability between its studies.

CSLT showed a significant effect in improving comprehension compared to controls (SMD = 1.23, 95% CI [0.57, 1.89], *p* < 0.001, *I*^2^ = 46%), suggesting that the effect of this therapy varied somewhat across studies, but the overall effect remained robust. In contrast, among other therapies, LCT significantly improved comprehension (SMD = 0.81, 95% CI [0.35, 1.27], *p* < 0.001, *I*^2^ = 31%), suggesting that the effect of this therapy was more consistent across studies. However, the effect of SLT on COMPREHENSION was not significant (SMD = 0.62, 95% CI [−0.33, 1.58], *p* > 0.05, *I*^2^ = 84%), suggesting that the results differed across studies.

CSLT demonstrated a significant effect in improving REPETITION compared to control (SMD = 1.22, 95% CI [0.03, 2.40], *p* < 0.05, *I*^2^ = 86%), suggesting that the effect of this therapy varied across studies. LCT significantly improved REPETITION (SMD = 0.51, 95% CI [0.01, 1.00], *p* < 0.05, *I*^2^ = 0%), indicating a highly consistent effect across studies. However, the effect of SLT on REPETITION was not significant (SMD = 0.86, 95% CI [−0.13, 1.85], *p* > 0.05, *I*^2^ = 85%), suggesting inconsistency between studies.

#### 3.4.2 Secondary outcomes

**Figure 6:**
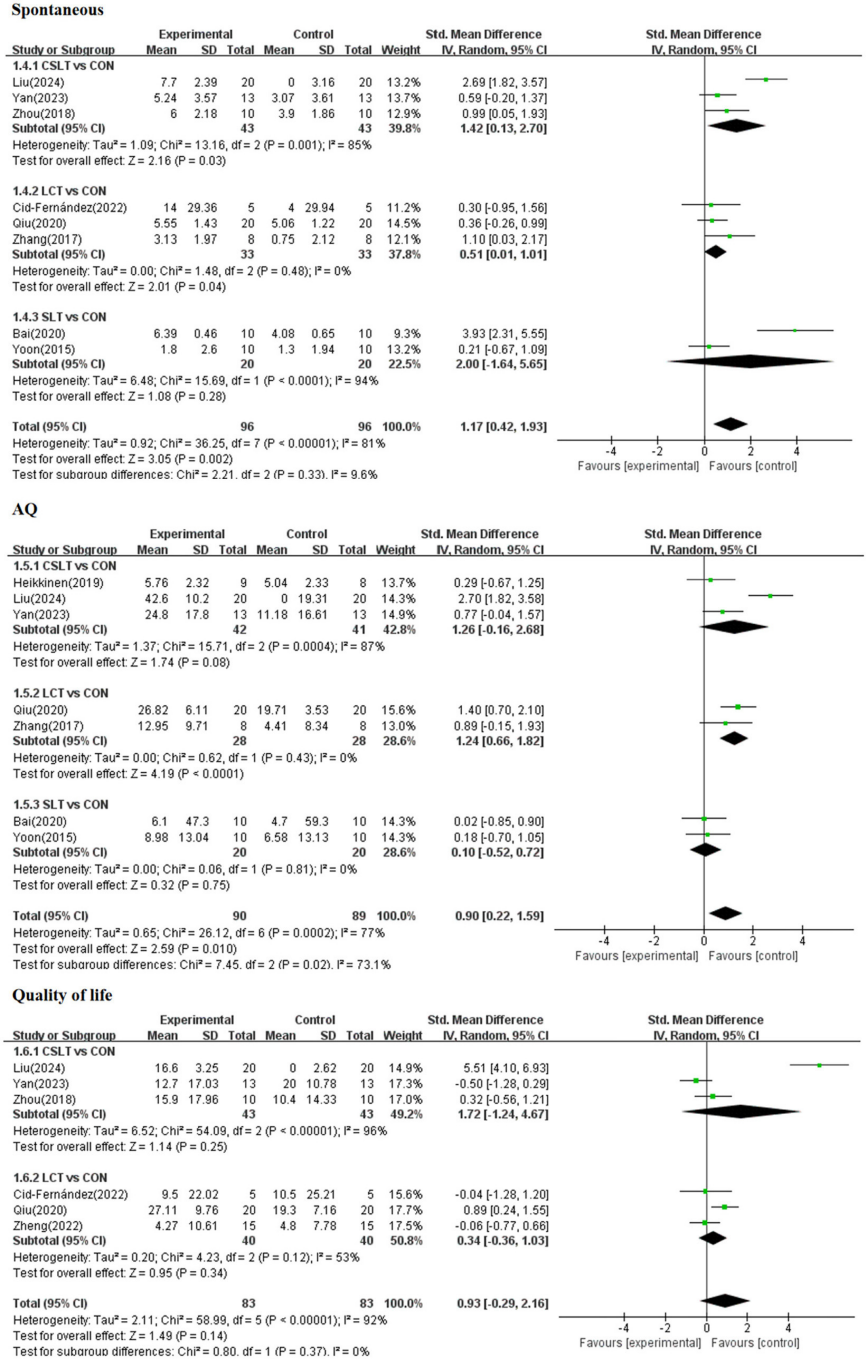
Forest plot of secondary outcomes.

A forest plot of secondary outcomes based on non-invasive treatments is shown in Figure 6. The results of the two-by-two meta-analysis showed that LCT demonstrated significant improvement in most outcomes. In particular, LCT had a significant effect on both AQ (SMD = 1.24, 95% CI [0.66, 1.82], *p* < 0.0001, *I*^2^ = 0%) and spontaneous (SMD = 1.42, 95% CI [0.13, 2.70], *p* < 0.05, *I*^2^ = 0%), with a high degree of consistency of the effect across studies. The effect of CSLT on the improving spontaneous (SMD = 1.42, 95% CI [0.13, 2.70], *p* < 0.05, *I*^2^ = 85%) but the results varied across studies. SLT had a significant effect on improving spontaneous (SMD = 2.00, 95% CI [−1.64, 5.65], *p* > 0.05, *I*^2^ = 94%) and AQ (SMD = 0.90, 95% CI [0.22, 1.59], *p* > 0.05, *I*^2^ = 0%).

### 3.5 Network meta-analysis

#### 3.5.1 Network diagram of included studies

**Figure 7:**
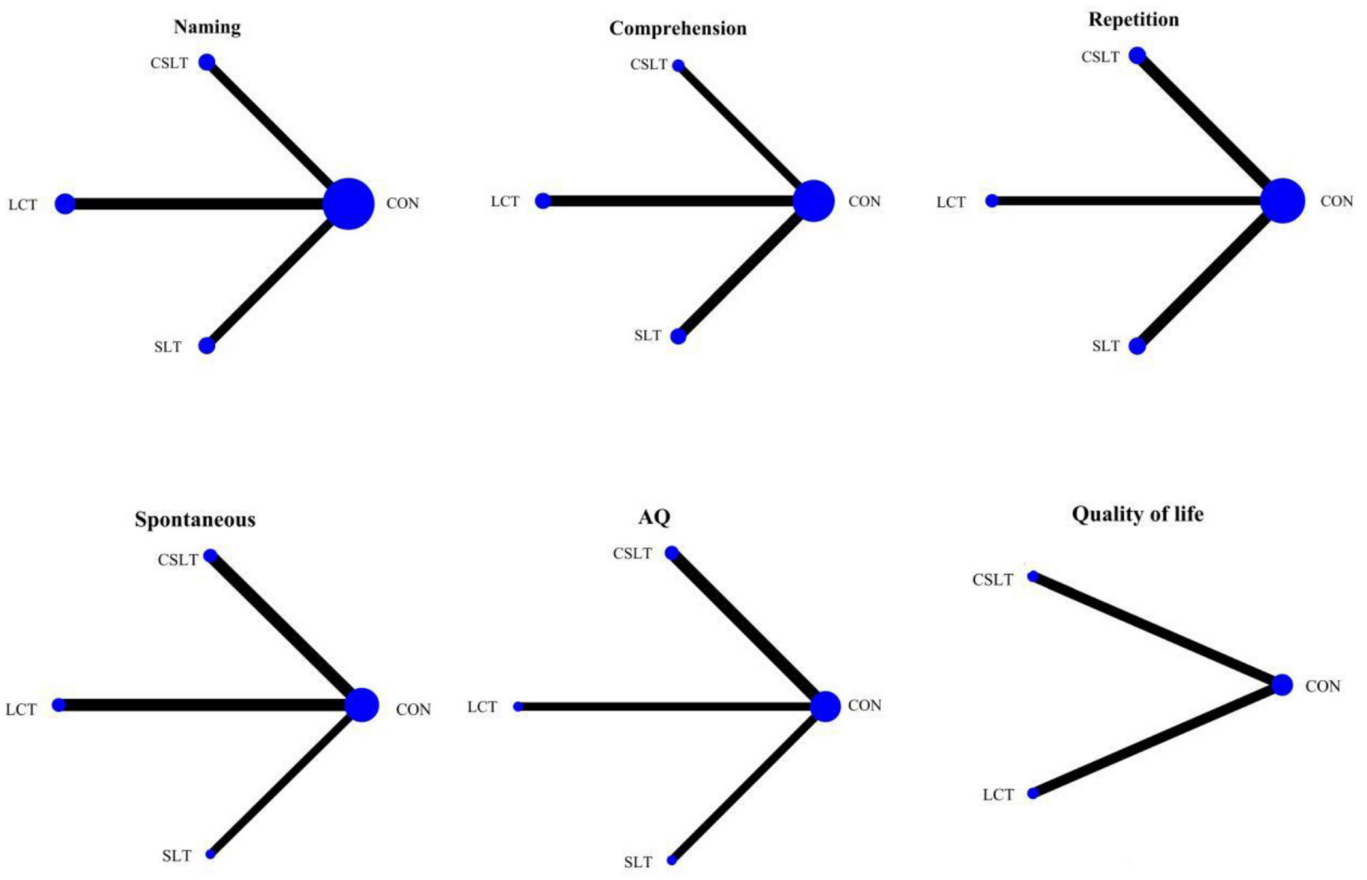
Network plot of outcome indicators.

The 4 dots in the figure represent the 4 interventions, the straight lines between the dots represent the existence of direct comparisons between interventions, and the thickness of the line represents the number of direct comparisons between the two interventions. Except for the quality-of-life indicator, which was 3 interventions, all outcome indicators were 4 interventions (including the control group) and included the same interventions. Interventions in the experimental group included CSLT, LCT, and SLT combined with transcranial electromagnetic stimulation; the control group was a no combined group, and LCT was the most widely studied intervention. The Network diagram of the outcome metrics is detailed in Figure 7.

#### 3.5.2 Summary estimates and ranking of intervention effectiveness of three rehabilitation methods combined with transcranial electromagnetic stimulation

**Figure 8:**
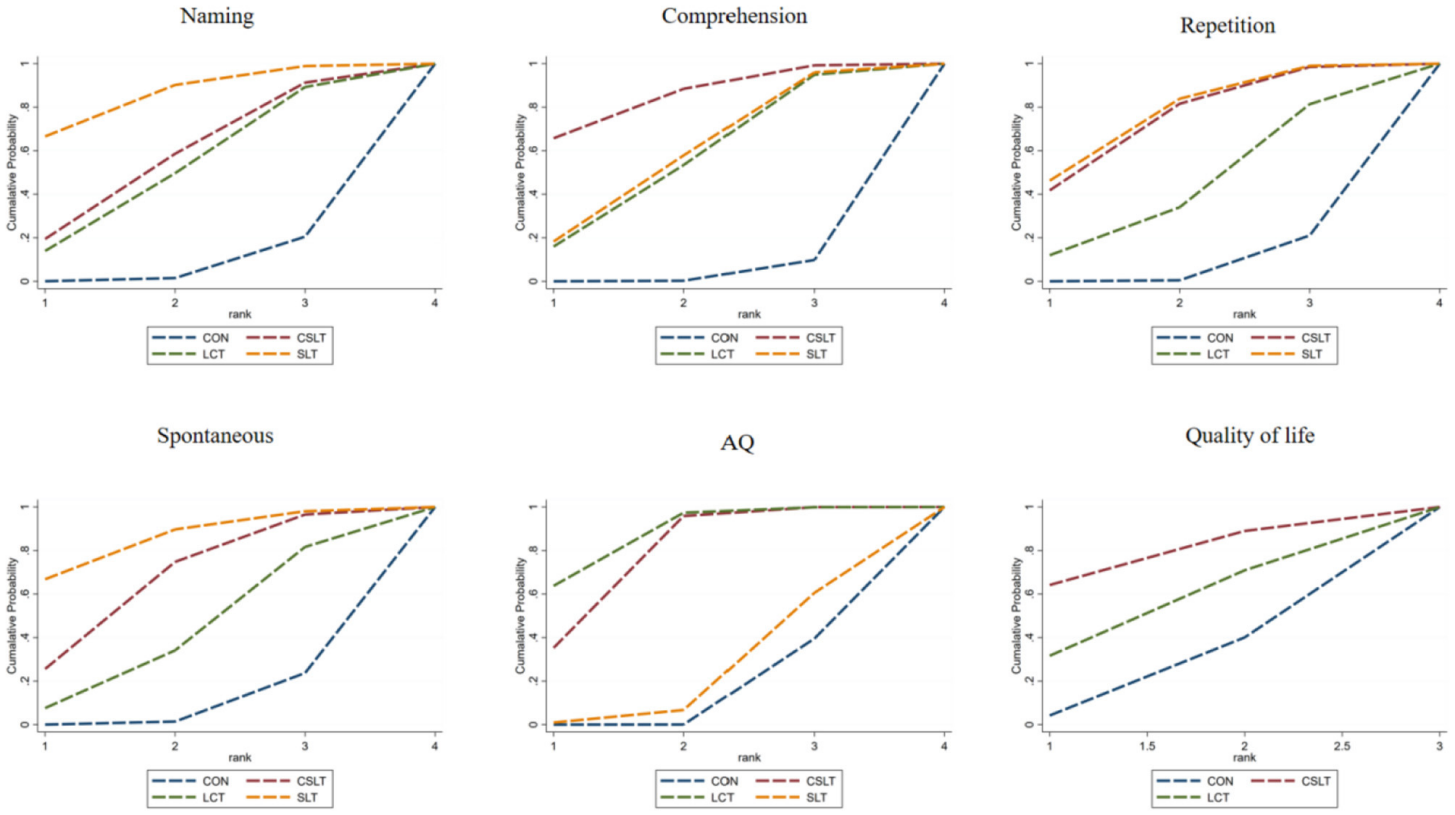
Ranking of intervention effects for outcome indicators.

Naming index: the effectiveness of the three rehabilitation methods combined with transcranial electromagnetic stimulation on aphasia in stroke patients was ranked as SLT (SMD = 1.09, 95% CI [0.16,2.02], *p* < 0.05, [SUCRA] = 85.2), CSLT (SMD = 0.62, 95% CI [−0.31,1.55], *p* > 0.05, [SUCRA] = 56.5), and LCT (SMD = 0.52, 95% CI [−0.34,1.37], *p* > 0.05, [SUCRA] = 51.0) were superior to the control group CON ([SUCRA] = 7.3) without transcranial electromagnetic stimulation. The improvement effect of SLT was significant, while the effect of other interventions was not statistically significant (see Figure 8 and Tables 2, 3).

**Table 2:** Ranking of the probability of improving aphasia-related indicators and quality of life class in stroke patients by three rehabilitation methods combined with transcranial electromagnetic stimulation.

**Treatment**	**Naming**		**Comprehension**		**Repetition**		**Spontaneous**		**AQ**		**Quality of life**	
	**SUCRA (%)**	**Rank**	**SUCRA (%)**	**Rank**	**SUCRA (%)**	**Rank**	**SUCRA (%)**	**Rank**	**SUCRA (%)**	**Rank**	**SUCRA (%)**	**Rank**
CON	7.3	4	3.4	4	7.2	4	8.4	4	13.2	4	22.1	3
CSLT	56.5	2	84.5	1	74.0	2	65.6	2	77.0	2	76.6	1
LCT	51.0	3	54.8	3	42.5	3	41.1	3	87.0	1	51.3	2
SLT	85.2	1	57.4	2	76.4	1	84.9	1	22.7	3		

Darker color means higher score or bigger ranking.

**Table 3:** Network meta-analysis matrix of outcome.

**Naming**	**Comprehension**						
SLT	−0.47 (−1.78, 0.84)	−0.57 (−1.83, 0.69)	−1.09 (−2.02, −0.16)	CSLT	−0.38 (−1.43, 0.66)	−0.42 (−1.48, 0.65)	−1.01 (−1.80, −0.22)
0.47 (−0.84, 1.78)	CSLT	−0.10 (−1.36, 1.15)	−0.62 (−1.55, 0.31)	0.38 (−0.66, 1.43)	SLT	−0.03 (−1.01, 0.95)	−0.63 (−1.30, 0.05)
0.57 (−0.69, 1.83)	0.10 (−1.15, 1.36)	LCT	−0.52 (−1.37, 0.34)	0.42 (−0.65, 1.48)	0.03 (−0.95, 1.01)	LCT	−0.59 (−1.30, 0.12)
1.09 (0.16, 2.02)	0.62 (−0.31, 1.55)	0.52 (−0.34, 1.37)	CON	1.01 (0.22, 1.80)	0.63 (−0.05, 1.30)	0.59 (−0.12, 1.30)	CON
**Repetition**	**Spontaneous**						
CSLT	0.04 (−1.01, 1.10)	−0.42 (−1.60, 0.76)	−0.83 (−1.58, −0.07)	SLT	−0.64 (−2.78, 1.50)	−1.20 (−3.36, 0.96)	−1.78 (−3.50, −0.06)
−0.04 (−1.10, 1.01)	SLT	−0.46 (−1.63, 0.71)	−0.87 (−1.61, −0.13)	0.64 (−1.50, 2.78)	CSLT	−0.56 (−2.38, 1.27)	−1.14 (−2.41, 0.13)
0.42 (−0.76, 1.60)	0.46 (−0.71, 1.63)	LCT	−0.41 (−1.31, 0.50)	1.20 (−0.96, 3.36)	0.56 (−1.27, 2.38)	LCT	−0.58 (−1.89, 0.72)
0.83 (0.07, 1.58)	0.87 (0.13, 1.61)	0.41 (−0.50, 1.31)	CON	1.78 (0.06, 3.50)	1.14 (−0.13, 2.41)	0.58 (−0.72, 1.89)	CON
**AQ**	**Quality of life**						
LCT	−0.19 (−1.24, 0.86)	−1.10 (−2.27, 0.07)	−1.20 (−2.02, −0.38)	CSLT	−0.38 (−2.12, 1.37)		−0.70 (−1.93, 0.53)
0.19 (−0.86, 1.24)	CSLT	−0.91 (−1.98, 0.15)	−1.01 (−1.68, −0.35)	0.38 (−1.37, 2.12)	LCT		−0.32 (−1.56, 0.92)
1.10 (−0.07, 2.27)	0.91 (−0.15, 1.98)	SLT	−0.10 (−0.93, 0.73)	0.70 (−0.53, 1.93)	0.32 (−0.92, 1.56)		CON

Comprehension index: The effectiveness of the three rehabilitation methods combined with transcranial electromagnetic stimulation on aphasia in stroke patients was ranked as CSLT (SMD = 1.01, 95% CI [0.22, 1.80], *p* < 0.05, [SUCRA] = 84.5), SLT (SMD = 0.63, 95% CI [−0.05,1.30], *p* > 0.05, [SUCRA] = 57.4), and LCT (SMD = 0.59, 95% CI [−0.12,1.30], *p* > 0.05, [SUCRA] = 54.8) were better than that of the control group without transcranial electromagnetic stimulation CON ([SUCRA] = 3.4). The improvement effect of CSLT was significant, while the effect of other interventions was not statistically significant (see Figure 8 and Tables 2, 3).

Repetition index: the effectiveness of the three rehabilitation methods combined with transcranial electromagnetic stimulation on aphasia in stroke patients was ranked as SLT (SMD = 0.87, 95% CI [0.13,1.61], *p* < 0.05, [SUCRA] = 76.4), CSLT (SMD = 0.83, 95% CI [0.07,1.58], *p* < 0.05, [SUCRA] = 74.0), and LCT (SMD = 0.41, 95% CI [−0.50,1.31], *p* > 0.05, [SUCRA] = 42.5) were superior to the control group CON without transcranial electromagnetic stimulation ([SUCRA] = 7.2). SLT and CSLT showed significant effects, while LCT showed some effects but did not reach statistical differences (see Figure 8 and Tables 2, 3 for details).

Spontaneous index: the effectiveness of the three rehabilitation methods combined with transcranial electromagnetic stimulation on aphasia in stroke patients was ranked as SLT (SMD = 1.78, 95% CI [0.06,3.50], *p* < 0.05, [SUCRA] = 84.9), CSLT (SMD = 1.14, 95% CI [−0.13,2.41], *p* > 0.05, [SUCRA] = 65.6), and LCT (SMD = 0.58, 95% CI [−0.72,1.89], *p* > 0.05,[SUCRA] = 41.1) were all better than the control group without transcranial electromagnetic stimulation CON ([SUCRA] = 8.4). SLT showed significant effect, while CSLT and LCT showed some effect but no statistical significance (see Figure 8 and Tables 2, 3 for details).

AQ index: the effectiveness of the three rehabilitation methods combined with transcranial electromagnetic stimulation on aphasia in stroke patients was ranked as LCT (SMD = 1.20, 95% CI [0.38, 2.02], *p* < 0.05, [SUCRA] = 87.0), CSLT (SMD = 1.01, 95% CI [0.35, 1.68], [SUCRA] = 77.0), and SLT (SMD = 0.10, 95% CI [−0.73, 0.93], *p* > 0.05, [SUCRA] = 22.7) were all superior to the control group CON without transcranial electromagnetic stimulation ([SUCRA] = 13.2). LCT and CSLT showed significant effects, while SLT did not show significant effects (see Figure 8 and Tables 2, 3).

Quality of life index: the effectiveness of the three rehabilitation methods combined with transcranial electromagnetic stimulation on aphasia in stroke patients was ranked as CSLT (SMD = 0.70, 95% CI [−0.53, 1.93], *p* > 0.05, [SUCRA] = 76.6) and LCT (SMD = 0.32, 95% CI [−0.92, 1.56], *p* > 0.05, [SUCRA] = 51.3) were better than the control group CON ([SUCRA] = 22.1) without transcranial electromagnetic stimulation. Both interventions were effective but there was no statistical difference (see Figure 8 and Tables 2, 3). The summary table of different interventions is shown in Table 4.

**Table 4:** Summary table.

**Group**	***k***	**Effectiveness**	**Usability**	**Acceptability**	**Recommendation level**
CSLT	5	Performs well on all indicators (top two), with the best effects observed on comprehension and quality of life.	High	High	1
LCT	5	Moderate effects on most indicators, but relatively good effects on AQ and quality of life.	High	Moderate	3
SLT	4	It performs well on most indicators, particularly excelling in naming, repetition, and spontaneous speech, but shows poorer results on the AQ measure.	High	Moderate	2

K represents the number of articles included, and the lower the recommendation grade number, the higher the grade.

### 3.6 Small-sample effects or publication bias tests

**Figure 9:**
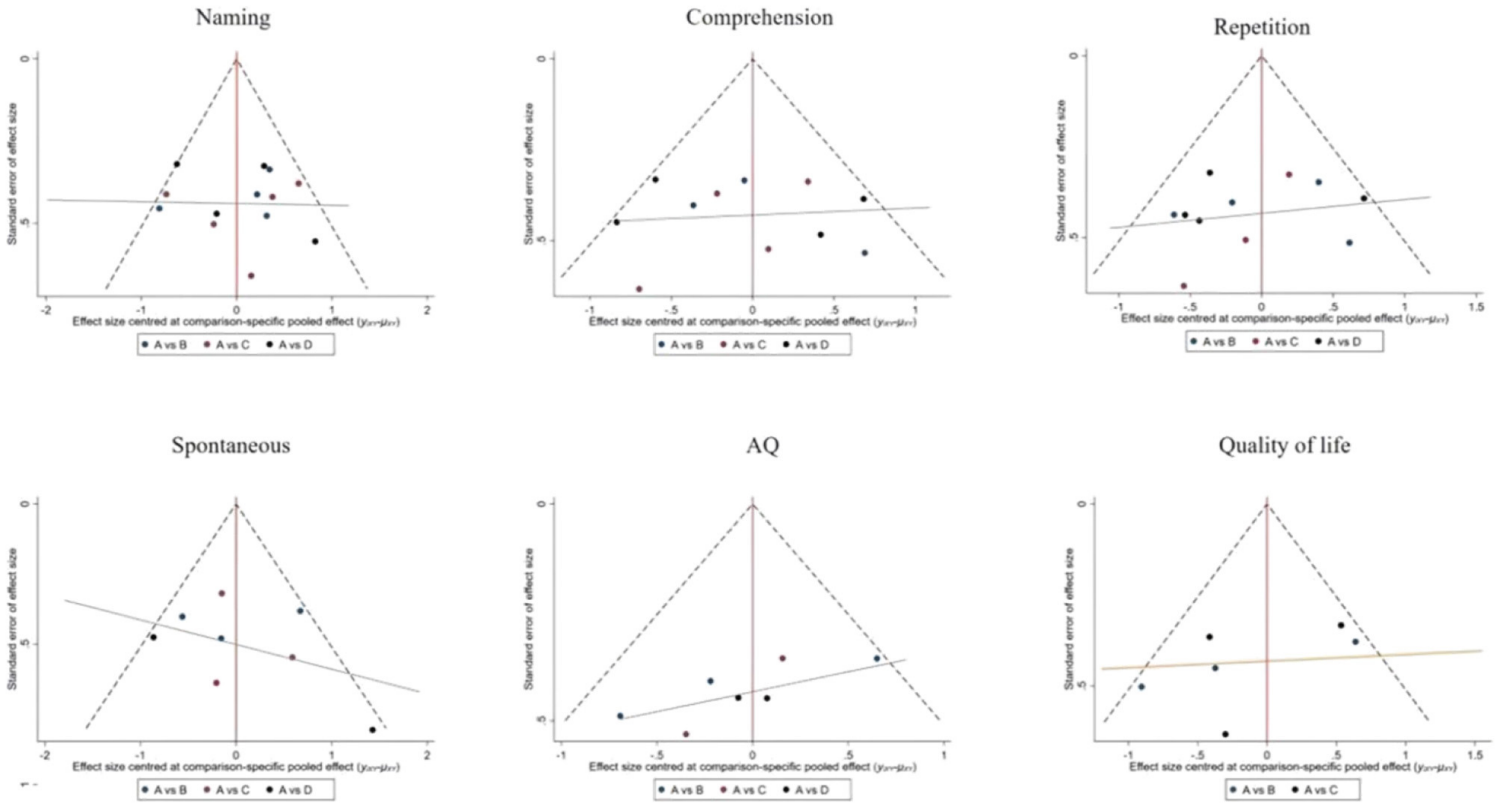
Corrected comparison funnel plot for outcome indicators. A = CON; B = CSLT; C = LCT; D = SLT.

For studies included in the reticulated META analysis, small-sample effect estimates and publication bias tests were performed using corrected-comparison funnel plots. The included studies were largely symmetrical, suggesting that there was no small-sample effect in the current study, and no significant publication bias was found (see Figure 9).

## 4 Discussion

### 4.1 Analysis of the efficacy of three non-invasive treatments on the main outcome indicators of aphasia in stroke patients

In this study, Naming, Comprehension and Repetition were analyzed as the main functional indicators. The results showed that there were significant differences in the efficacy of the three treatments on different functional indicators, and their respective effects showed different study heterogeneity. First, on the Naming metric, both LCT and SLT showed significant improvement effects, with SMDs of 1.54 (95% CI [0.04, 2.64], *p* < 0.05) for LCT and 1.05 (95% CI [0.45, 1.65], *p* < 0.001) for SLT, whereas the effect of CSLT failed to reach a significant level (SMD = 0.85, *p* > 0.05). This result suggests that although CSLT failed to produce a significant effect in Naming, LCT and SLT have better results in the treatment of aphasia, which is consistent with previous studies, and the Schaffer et al.'s (20) study, which also found that cognitive-behavioral therapy had a positive effect on several language outcomes. SLT, on the other hand, is widely used for speech recovery in aphasia patients (21). Therefore, we can hypothesize that these therapies may affect the recovery of naming ability through different mechanisms, but the differences in responses of different individuals may lead to inconsistent efficacy. Second, in terms of Comprehension, CSLT showed significant improvement (SMD = 1.23, 95% CI [0.57, 1.89], *p* < 0.001), while LCT (SMD = 0.81, 95% CI [0.35, 1.27], *p* < 0.001) and SLT (SMD = 0.62, 95% CI [−0.33, 1.58], *p* > 0.05) were weaker, with SLT failing to even reach statistical significance. This result is consistent with the results of several studies, such as Wang et al.'s (22) study, which showed the high effectiveness of CSLT in language comprehension recovery. Whereas, the non-significant effect of SLT on Comprehension may be related to individual differences in the specific approach and implementation of language comprehension in the treatment process. In terms of Repetition, both CSLT (SMD = 1.22, 95% CI [0.03, 2.40], *p* < 0.05) and LCT (SMD = 0.51, 95% CI [0.01, 1.00], *p* < 0.05) significantly improved the patients' repetition ability, however, the effect of SLT was not significant (SMD = 0.86, 95% CI [−0.13, 1.85], *p* > 0.05). This result suggests that LCT and CSLT have a potential advantage in restoring repetition ability in patients with aphasia, whereas the effect of SLT is more limited. In line with our findings, the literature shows that Simic et al. (23) demonstrated that cognitive training enhances the effectiveness of speech therapy and improves repetition ability and communication outcomes. In contrast, the effect of SLT on repetition ability is dependent on the individual's stage of rehabilitation and the continuity of treatment.

### 4.2 Analysis of the efficacy of three non-invasive treatments on secondary outcome indicators of aphasia in stroke patients

In this study, Spontaneous, AQ and Quality of life were analyzed as the secondary indicators. On the Spontaneous index, the effectiveness rankings of the three combined treatments were, in order, SLT ([SUCRA] = 84.9), CSLT ([SUCRA] = 65.6), and LCT ([SUCRA] = 41.1), all of which were significantly better than that of the control CON ([SUCRA] = 8.4).

LCT treatment showed the most significant performance in improving AQ metrics ([SUCRA] = 87.0), which was superior to SLT and CSLT, and LCT also showed a better effect on Quality of life scores ([SUCRA] = 51.3). Further analyses showed that LCT demonstrated more consistent and significant improvements in multiple outcomes, particularly statistically significant in AQ (SMD = 1.24, 95% CI [0.66, 1.82], *p* < 0.0001) and Spontaneous (SMD = 1.42, 95% CI [0.13, 2.70], *p* < 0.05). This is consistent with previous findings, where Haghighi et al. (24) showed that cognitive training enhances the effectiveness of speech therapy and improves language and communication outcomes.

However, the effect of CSLT on Spontaneous was more prominent, but there was greater between-study heterogeneity (*I*^2^ = 85%), which, when compared to LCT, may indicate that the effect of CSLT varies across populations or study settings. SLT, although it also showed a trend toward improvement in Spontaneous and AQ, did not achieve statistical significance, particularly in Spontaneous (SMD = 2.00, 95% CI [−1.64, 5.65], *p* > 0.05) and AQ (SMD = 0.90, 95% CI [0.22, 1.59], *p* > 0.05) where the differences failed to fall outside of random fluctuations, which is in line with the findings of some studies, Haghighi et al.'s (24) study found significant improvements in content, fluency, and aphasia quotient, with smaller effect sizes on other indicators of speech function. It may reflect a potential limitation of the SLT approach when combined with transcranial electromagnetic stimulation.

### 4.3 Critical analysis

As can be seen from the above analysis, LCT shows the most remarkable performance in improving AQ and spontaneous speech, which may be related to its comprehensive training mechanism that can simultaneously activate multiple brain regions and promote the overall recovery of language functions. However, the advantage of CSLT in comprehension indicates its strong targeting in specific language functions and suggests that it may be more suitable for patients with comprehension impairments. Although SLT failed to reach significance in some indicators, its widespread clinical application and potential contribution to speech recovery should not be overlooked, especially when combined with individualized treatment plans. Future research should further explore the applicability of these techniques in different aphasia subtypes and rehabilitation stages, as well as how to optimize combined treatment protocols to enhance therapeutic effects. In addition, considering that SLT has not shown significant effects in some studies, it may be necessary to re-evaluate its application conditions in specific populations and how to enhance its efficacy by improving treatment procedures or increasing treatment intensity.

### 4.4 Limitation

This study has several limitations. The quality and sample sizes of the included studies varied, which may affect the robustness of the findings. Additionally, the heterogeneity in study designs and the short follow-up periods limit the generalizability and long-term conclusions. Currently, there are relatively few studies on combined treatments for post-stroke aphasia, which further highlights the need for more comprehensive research in this area.

Future research should focus on conducting large-scale RCTs with standardized protocols and longer follow-up periods to provide more reliable evidence. Exploring the underlying mechanisms and developing personalized treatment plans based on individual patient characteristics are also important directions for improving post-stroke aphasia rehabilitation.

## 5 Conclusion

SLT was most effective in improving Naming, Repetition and Spontaneous, but in CSLT was most effective in improving Comprehension and Quality of life in stroke patients, and LCT was most effective in improving AQ. These results suggest that SLT and CSLT have significant advantages in improving aphasia in stroke patients, pointing to the potential value of both interventions in clinical practice and suggesting that they should be used as preferred intervention options in clinical practice. The combination of different interventions should be individualized based on the patient's specific dysfunction and physical condition to promote functional recovery. Future studies should further explore the long-term effects of these interventions and search for optimal parameter configurations.

## Data Availability

The original contributions presented in the study are included in the article/supplementary material, further inquiries can be directed to the corresponding author.
